# Ischemic Preconditioning Provides Neuroprotection by Inhibiting NLRP3 Inflammasome Activation and Cell Pyroptosis

**DOI:** 10.3390/brainsci13060897

**Published:** 2023-06-01

**Authors:** Li Gao, Xin Sun, Meibo Pan, Wenrui Zhang, Desheng Zhu, Zhongjiao Lu, Kan Wang, Yinfeng Dong, Yangtai Guan

**Affiliations:** 1 Department of Neurology, Ren Ji Hospital, Shanghai Jiao Tong University School of Medicine, Shanghai 200127, China; 2 Department of Pharmacology, School of Basic Medical Science, Nanjing Medical University, Nanjing 211166, China; 3 Department Pathology and Pathophysiology, School of Medicine & Holistic Integrative Medicine, Nanjing University of Chinese Medicine, Nanjing 210023, China; 4 Department of Neurosurgery, Ren Ji Hospital, Shanghai Jiao Tong University School of Medicine, Shanghai 200127, China

**Keywords:** ischemic preconditioning (IPC), NLRP3 inflammasome, pyroptosis, ischemic stroke, neuroprotection

## Abstract

Increasing evidence has demonstrated that ischemic preconditioning (IPC) increases cerebral tolerance to subsequent prolonged ischemic insults. However, the exact mechanisms underlying the process have not been fully explored. In the current study, we aim to investigate whether NLRP3 inflammasome and cell pyroptosis are involved in the neuroprotective mechanism of IPC after ischemic stroke. In vitro, IPC was set up by exposing BV-2 cells to 10 min of oxygen–glucose deprivation (OGD). In vivo, IPC was performed by a transient cerebral ischemia of 10 min occlusion of the middle cerebral artery (MCA) in mice. We found that the NLRP3 inflammasome was activated and cell pyroptosis was induced at 6 h and 24 h post-stroke in an ischemic brain. IPC treatment increased cell viability under OGD state, reduced the infarct size, and attenuated the neurological deficits of mice. However, the effects NLRP3 inflammasome activation and pyroptosis after stroke were attenuated by IPC, which decreased the expression of NLRP3, ASC, cleaved caspase 1, and GSDMD-N and reduced the production of IL-1β and IL-18. In addition, confocal immunofluorescence staining of Annexin V-mCherry and SYTOX green was inhibited by IPC. These findings suggest a more enhanced link between IPC and inflammatory signature and cell death, highlighting that the NLRP3 inflammasome may act as a promising target for the prevention and treatment of ischemic stroke.

## 1. Introduction

Globally, stroke has been reported as the leading cause of mortality and long-term disability. Over the past 20 years, limitations of reperfusion therapy in clinical application and recurrent failures in neuroprotectants highlight the need to develop new therapeutic strategies for the treatment of ischemic stroke. Ischemic preconditioning (IPC), referred to as nonlethal transit ischemia, could confer endogenous neuroprotection against subsequent lethal ischemic damage [1]. To date, the beneficial effects of IPC have been successfully verified in experimental studies and some clinical trials [2,3,4]. However, the exact mechanisms underlying the process remain unknown. 

Inflammasomes are large multimolecular complexes that function as intracellular sensors for infectious agents and host-derived danger signals that are associated with neurological diseases [5]. The inflammasomes generally comprise three domains: a cytosolic pattern-recognition sensor, an adaptor protein, and an effector protease caspase-1. Nod-like receptor protein 3 (NLRP3) is the most well-characterized sensor that contains an N-terminal pyrin domain (PYD), a central nucleotide-binding domain (NACHT), and a C-terminal leucine-rich repeats (LRRs) domain. In response to microbial infection and cellular damage, NLRP3 recruits the adaptor apoptosis-associated speck-like protein containing a CARD (ASC), which interacts with the effector pro-caspase-1. Then, pro-caspase-1 is cleaved and mediates caspase-1 activation, in turn, regulating the secretion of proinflammatory cytokines interleukin-1β (IL-1β) and IL-18. Additionally, the cleaved caspase-1 can proteolytically cleave gasdermin D (GSDMD) to form membrane pores for cytokine release and induce pyroptosis [6]. Anomalous NLRP3 inflammasome activity has been reported to be associated with many diseases, including neurodegenerative diseases, autoimmune diseases, and cardiovascular diseases [7,8,9].

The NLRP3 inflammasome is abundantly expressed in monocyte, neutrophils, microglia, and dendritic cells, which is crucial to immune defenses and neuroinflammatory responses [10]. Recently, increasing evidence has suggested that the NLRP3 inflammasome play a vital role in detecting cellular damage and mediating neuroinflammation during ischemic stroke [11,12,13]. Direct targeting of the NLRP3 inflammasome may provide neuroprotection and salvage neurological deterioration [14,15]. The endogenous protective effects and the relationship between IPC and the NLRP3 inflammasome after ischemic stroke have not been elucidated. In the present study, we established an IPC model in vivo and in vitro to explore whether the NLRP3 inflammasome and pyroptosis are involved in the neuroprotective process of IPC under ischemic conditions. 

## 2. Materials and Methods

### 2.1. Animals 

Male C57BL/6 mice (10–12 weeks old) were purchased from Nanjing Medical University. All animal experiments and procedures were approved by the Institutional Animal Care and Use Committee of Nanjing Medical University, Nanjing, China. Reporting of all animal studies complied with the ARRIVE guidelines. The mice were bred and housed in a standard animal room with a 12 h day and 12 h night cycle; the mice were able to consume food and water freely. 

### 2.2. Middle Cerebral Artery Occulusion Model (MCAO) Establishment

**Figure 1:**
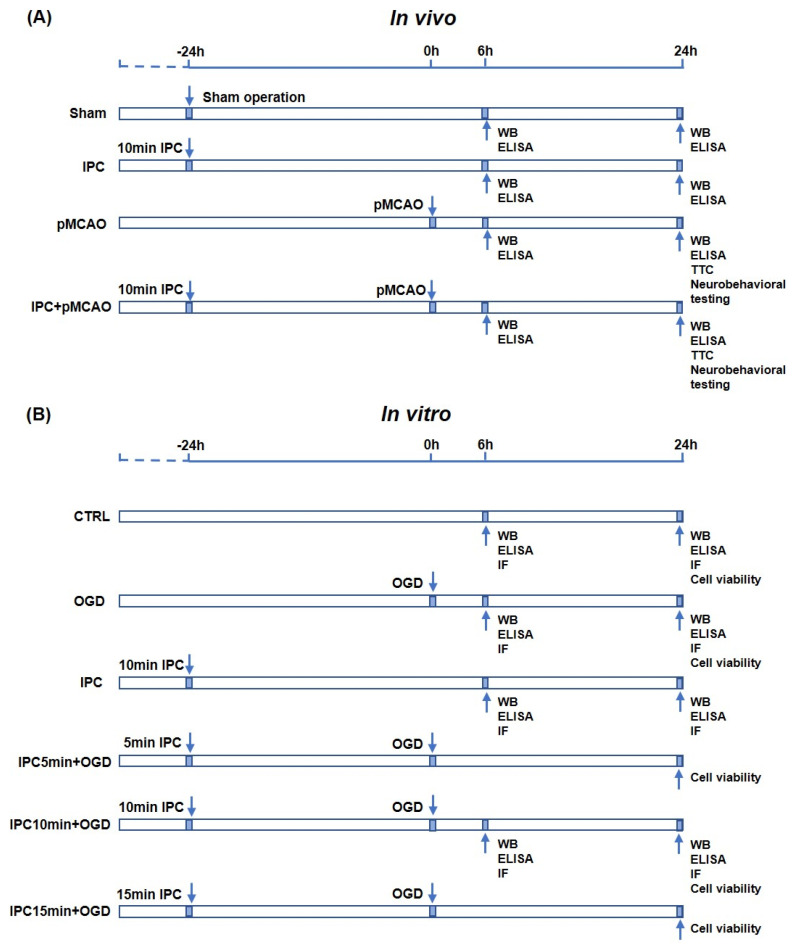
Scheme of the main experimental protocols in vivo and vitro. (**A**) The in vivo experimental protocol. (**B**) The in vitro experimental protocol. IPC: ischemic conditioning; pMCAO: permanent middle cerebral artery occlusion; OGD: oxygen−glucose deprivation; WB: Western blotting; ELISA: enzyme-linked immunosorbent assay; IF: immunofluorescence staining.

The permanent focal cerebral ischemia (permanent middle cerebral artery occlusion, pMCAO) model was induced as described previously [16]. First, animals were anesthetized using 2–5% isoflurane inhalation. A midline incision was made in the neck, and a 6–0 silicone-coated nylon suture (6023PK, Doccol Corp, CA, USA) was inserted into the right middle cerebral artery (MCA) through the external carotid artery (ECA) incision. IPC was performed with 1 cycle transient focal ischemia of 10 min occlusion. After IPC induction, the inserted suture was withdrawn to permit reperfusion, the ECA was ligated, and the neck wound was carefully closed. Then, 24 h later, the suture was reintroduced into MCA through the ECA incision to induce permanent focal cerebral ischemia. For the IPC group, the mice only underwent transient focal ischemia of 10 min, whereas the pMCAO group underwent permanent focal cerebral ischemia surgery without prior IPC. The sham group underwent identical surgery except for the suture insertion. Cerebral blood flow (CBF) was monitored with laser Doppler flowmetry (Moor VSM-LDF, Wilmington, DE, USA), and successful occlusion was marked by a reduction of more than 80% of CBF. A heating pad was applied to maintain body temperature of the mice at 37 °C during surgery. All animals were allowed ad libitum access to water and food after surgery. The detailed in vivo experimental protocol was indicated in Figure 1A.

### 2.3. Neurobehavioral Evaluation

After 24 h post-MCAO, the neurological deficits were assessed using a five-point scale by investigators blinded to the experimental design. In brief, the neuroscores were as follows: 0, no neurological deficits; 1, difficulty fully extending the left forelimb when pulling the tail; 2, circling to the left side when walking; 3, rolling to the left side; 4, no autonomous motor activity and unconsciousness.

### 2.4. Infarct Volume Assessment

2,3,5-Triphenyltetrazolium Chloride (TTC) staining was conducted to evaluate the infarct volume. The mice were anaesthetized and decapitated 24 h later. Their brains were collected and then sliced into five 1 mm-thick coronal sections. The brain slices were incubated in 2% TTC for 30 min at 37 °C in the dark. Afterwards, 4% paraformaldehyde was used to fix the sections overnight. The infarct volume was analyzed by Image J program.

### 2.5. Cell Cultures

BV-2 cells were generated by immortalizing primary murine microglial cells with a v-raf/v-myc oncogene–carrying retrovirus. The BV-2 cells were cultured in Dulbecco’s modified Eagle’s medium (DMEM) containing 10% fetal bovine serum (FBS) (GIBCO, Carlsbad, CA, USA), penicillin (100 U/mL), and streptomycin (100 mg/L). The cells were gently suspended in DMEM and plated in 6–well plates at a density of 1 × 10^5^ cells/mL. The experimental groups were divided as follows: (1) control group (CTRL), (2) IPC group, (3) OGD group, (4) IPC + OGD group.

### 2.6. Oxygen-Glucose Deprivation (OGD) and IPC Induction

OGD was conducted as previously reported [17]. In brief, the BV-2 cells were washed twice with glucose-free DMEM and then immersed in DMEM without glucose and FBS in an aerobic chamber. IPC insult was produced by exposing the cells to a brief duration of OGD, and then the cells were transferred to normoxic conditions. For the induction of IPC + OGD, IPC was conducted first with transit OGD. Then, the cells were kept for 24 h under normoxic conditions. After 24 h, they were exposed to 3 h of OGD as lethal ischemic insult. Afterwards, the culture medium was replaced with normal culture and transferred to the incubator for 6 h or 24 h for reoxygenation. In order to explore the paradigm of IPC stimuli in vitro, a relatively short duration of 5 min, 10 min, and 15 min for OGD was used as IPC, respectively. For the control group, the cells were cultured with normal cell medium under normoxic conditions. The detailed in vitro experimental protocol is indicated in Figure 1B.

### 2.7. Cell Viability Analysis

MTT assays were performed to assess cell viability. Briefly, BV-2 microglia were seeded at a density of 5 × 10^4^ cells per well and received IPC, OGD, or IPC + OGD treatment. After reoxygenation for different time courses, the MTT (0.5 mg/mL, Beyotime, Shanghai, China) was dissolved in the cell medium and incubated at 37 °C for 4 h. The absorbance at 570 nm was read by Dynatech MR5000 plate counter.

### 2.8. Western Blotting (WB)

For WB analysis, the mice were sacrificed and the peri-infarct tissues or the corresponding region of sham-operated mice were collected after 6 or 24 h of IPC and/or IPC + MCAO. Brain tissues were homogenized, and the total proteins were extracted. The cytosolic protein in samples was also extracted at 6 or 24 h after IPC and/or OGD. The protein concentration was determined with the Micro BCA Kit (Pierce Biotechnology, Rockford, IL, USA). The protein samples (50 μg) were subjected to 8–12% Tris-glycine SDS-PAGE and transferred to PVDF membranes with the electrophoretic transfer system. The membranes were then blocked with Tris-HCL buffer saline containing 10% skim milk and 0.1% Tween-20 (TBS-T) for 1 h at room temperature. Then, the membranes were incubated with primary antibody against NLRP3 (1:1000, #768319, Novus Biologicals, Centennial, CO, USA), ASC (1:1000, sc-514414, Santa Cruz, CA, USA), cleaved caspase-1 (1:1000, #AG-20B-0042-C, Adipogen, San Diego, CA, USA), and GSDMD-N (1:1000, #10137, Cell Signaling Technology, Danvers, MA, USA). Subsequently, the membranes were washed in TBS-T and incubated with secondary antibodies for 1 h at room temperature. Finally, immunoblots were scanned and analyzed via Image J program and normalized to a loading control β-actin (1:1000, #4970, Cell Signaling Technology, USA).

### 2.9. Enzyme-Linked Immunosorbent Assay (ELISA)

The serum of the mice and the supernatant of the cells was collected. The total protein was quantitated and the production of the inflammatory cytokines IL-1β and IL-18 were detected with specific ELISA kit for IL-1β (R&D Systems, Minneapolis, MN, USA) and IL-18 (R&D Systems, USA), used in accordance with the manufacturers’ instructions.

### 2.10. Immunofluorescence Staining

To determine whether pyroptosis was involved in the neuroprotection of IPC, the double staining of Annexin V-mCherry and SYTOX green (Beyotime, Shanghai, China) was assessed. After 6 or 24 h following IPC and/or OGD, the BV-2 cells were harvested and seeded, at a density of 5 × 10^4^ cells per well, onto 24-well plates. After being centrifuged at 1000 rpm for 5 min, the cell culture medium was removed and the cells were rinsed with PBS. Then, Annexin V-mCherry binding buffer (194 μL) together with Annexin V-mCherry (5 μL) and SYTOX green (1 μL) was added. After incubation for 20 min at 20 °C in dark, the cells were washed and imaged to determine their fluorescence levels with a confocal microscope (Leica, Wetzlar, Germany). Positive cells were identified by colocalization with the Annexin V–mCherry Red and SYTOX green.

### 2.11. Statistical Analysis

Statistical analysis was performed by GraphPad Prism 8.0 software. For comparisons between two groups, Student’s *t*-test or the Mann-Whitney test were used to determine differences according to the statistical distribution. One-way analysis of variance (ANOVA) followed by the Student-Newman–Keuls (SNK) test were used to determine the differences involving three or more groups. The repeated measures were analyzed by two-way repeated measure ANOVA. *p* < 0.05 was considered as statistically significant. 

## 3. Results

### 3.1. IPC Could Attenuate Acute Ischemic Injury In Vivo and In Vitro 

**Figure 2:**
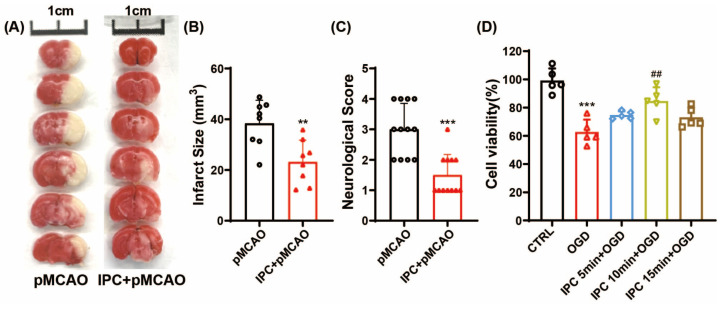
IPC attenuated acute ischemic injuries in vivo and in vitro under ischemic state. (**A**) TTC staining of representative brain sections at 24 h post–stroke in pMCAO and IPC + pMCAO groups. (**B**) Infarct volume was analyzed at 24 h following ischemic stroke (*n* = 8, ** *p* < 0.01 vs. pMCAO group). (**C**) The neurological score was assessed at 24 h after stroke (*n* = 12, *** *p* < 0.001 vs. pMCAO group). (**D**) Cell viability was assessed by MTT in different durations of IPC under OGD state (*n* = 5, *** *p* < 0.001 vs. CTRL group, ^##^ *p* < 0.01 vs. OGD group). TTC: 2,3,5-Triphenyltetrazolium Chloride; IPC: ischemic preconditioning; pMCAO: permanent middle cerebral artery occlusion; OGD: oxygen–glucose deprivation.

To determine the beneficial effects of IPC, the infarct volume and neurological deficit score of mice were assessed at 24 h after ischemic stroke. Consistently, we found IPC treatment significantly reduced infarct volume when compared to the pMCAO group (Figure 2A,B, ** *p* < 0.01 vs. pMCAO group). Additionally, IPC displayed a significantly decreased neurological score (Figure 2C, *** *p* < 0.001 vs. pMCAO group), suggesting that IPC could significantly mitigate cerebral ischemic injuries at 1 d post-stroke in vivo.

To explore the paradigms of IPC stimuli in vitro, IPC was performed by exposing BV-2 cells to OGD for 5 min, 10 min, and 15 min, respectively. As indicated in Figure 2D, the cell viability decreased by 25.4% after 3 h of OGD when compared to the control group (*** *p* < 0.001 vs. CTRL group). A different time duration of IPC could increase the cell viability to some extent, especially in the group treated with IPC for 10 min (^##^ *p* < 0.05 vs. OGD group). Subsequently, IPC was conducted for 10 min in the following experiments.

### 3.2. IPC Inhibited the Activated NLRP3 Inflammasome and Attenuated Inflammation in MCAO Mice 

**Figure 3:**
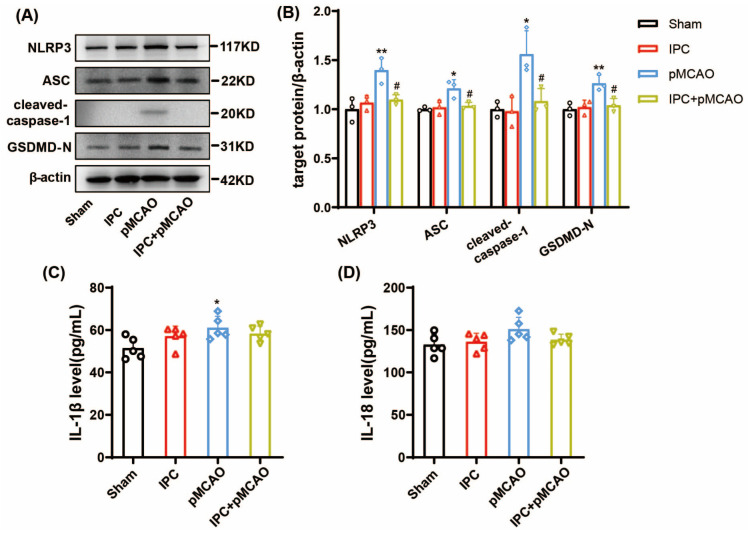
IPC inhibited the activation of NLRP3 inflammasome in mice at 6 h post-MCAO. (**A**,**B**) The protein level of NLRP3, ASC, cleaved-caspase-1, and GSDMD-N at 6 h in Sham, IPC, pMCAO, and IPC + pMCAO groups (*n* = 3, * *p* < 0.05, ** *p* < 0.01 vs. Sham group; ^#^ *p* < 0.05 vs. pMCAO group). (**C**,**D**) The production of IL-1β and IL-18 was analyzed with ELISA at 6 h after stroke in 4 groups (*n* = 5, * *p* < 0.05 vs. Sham group).

To explore the effects of IPC on NLRP3 inflammasome activation during ischemic stroke, we detected the protein level of NLRP3 components in the penumbra of mice. As indicated in Figure 3A,B, the expression of NLRP3, ASC, and cleaved-caspase-1 significantly elevated at 6 h post-stroke (* *p* < 0.05, ** *p* < 0.01 vs. Sham group), while IPC treatment could partially reverse it (^#^ *p* < 0.05 vs. pMCAO group). Further, GSDMD-N, the downstream target of cleaved-caspase-1, significantly increased at 6 h after stroke as well (** *p* < 0.01 vs. Sham group), while IPC inhibited stroke-induced upregulation of it (^#^ *p* < 0.05 vs. pMCAO group). Furthermore, we detected the content of inflammatory cytokines IL-1β and IL-18 in the serum of mice. It is shown in Figure 3C that IL-β increased after stroke when compared with the sham group (* *p* < 0.05 vs. Sham group). After treatment with IPC, the difference was not significant. As for IL-18, it tended to increase after ischemic stroke, but no significant difference was observed between the pMCAO group and the sham group (Figure 3D). These results indicate that IPC inhibits NLPR3 inflammasome activation at 6 h after ischemic stroke. 

**Figure 4:**
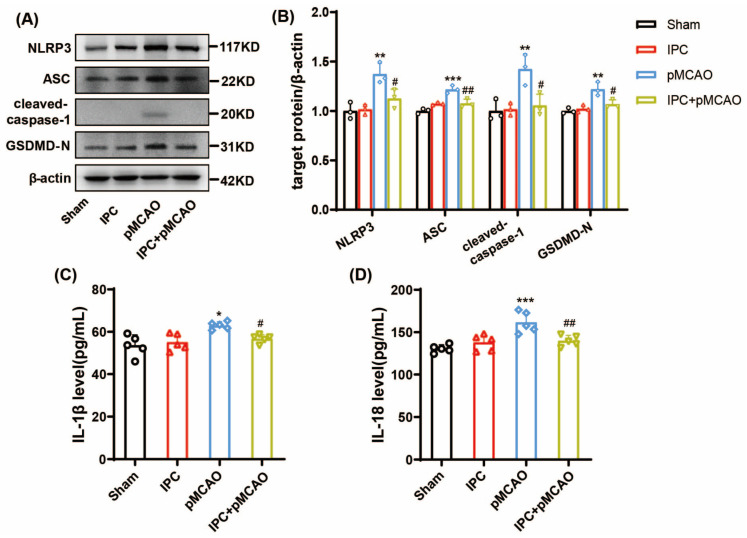
IPC inhibited the activation of NLRP3 inflammasome and inflammatory responses in mice at 24 h post-MCAO. (**A**,**B**) The protein level of NLRP3, ASC, cleaved caspase-1, and GSDMD-N at 24 h in Sham, IPC, pMCAO, and IPC + pMCAO groups (*n* = 3, ** *p* < 0.01, *** *p* < 0.001 vs. Sham group; ^#^ *p* < 0.05, ^##^ *p* < 0.01 vs. pMCAO group). (**C**,**D**) The production of IL-1β and IL-18 was assessed by ELISA at 24 h post-MCAO in 4 groups (*n* = 5, * *p* < 0.05, *** *p* < 0.001 vs. Sham group; ^#^ *p* < 0.05, ^##^ *p* < 0.01 vs. pMCAO group).

To further determine the time course of NLRP3 inflammasome activation and inflammatory reaction, we investigated the changes in NLRP3 inflammasome and inflammatory cytokines at 24 h following ischemic stroke. Consistent with the data shown at 6 h post-stroke, there was significantly upregulated expression of NLRP3, ASC, cleaved-caspase-1, and its downstream target GSDMD-N in the penumbra at 24 h after stroke (Figure 4A,B, ** *p* < 0.01, *** *p* < 0.001 vs. Sham group), while it could be partially reversed by the induction of IPC (^#^ *p* < 0.05, ^##^ *p* < 0.01 vs. pMCAO group). Similarly, the IL-1β and IL-18 serums showed a significant increase at 24 h after ischemic stroke (Figure 4C,D for IL-1β, * *p* < 0.05 vs. Sham group; for IL-18, *** *p* < 0.001 vs. Sham group). However, both IL-1β and IL-18 decreased significantly after the performance of IPC (Figure 4C,D, for IL-1β,^#^ *p* < 0.05 vs. pMCAO group; for IL-18, ^##^ *p* < 0.01 vs. pMCAO group). These findings show that IPC treatment could inhibit NLPR3 inflammasome activation and inflammatory responses at 24 h post-MCAO.

### 3.3. IPC Could Effectively Reduce NLRP3 Inflammasome Activation after OGD

**Figure 5:**
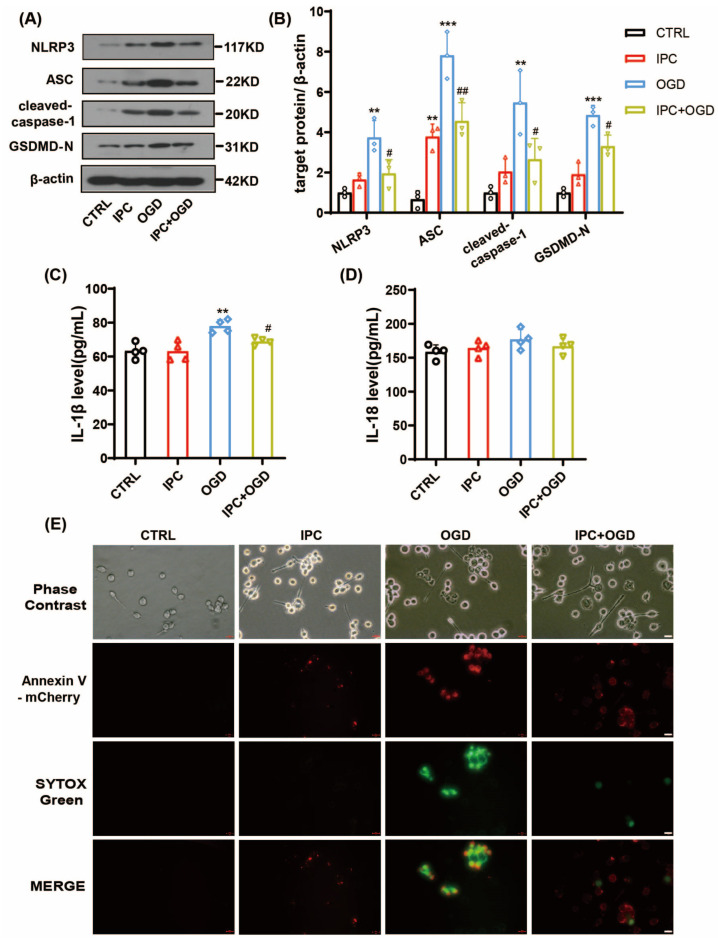
IPC reduced the activation of NLRP3 inflammasome and cell pyroptosis at 6 h in BV-2 cells under OGD state. (**A**,**B**) The expression of NLRP3, ASC, cleaved-caspase-1, and GSDMD-N at 6 h in control, IPC, OGD, and IPC + OGD groups (*n* = 3, ** *p* < 0.01, *** *p* < 0.01 vs. CTRL group; ^#^ *p* < 0.05, ^##^ *p* < 0.01 vs. OGD group). (**C**,**D**) The production of IL-β and IL-18 was assessed by ELISA at 6 h in BV-2 cells of the 4 groups (*n* = 4, ** *p* < 0.01 vs. CTRL group; ^#^ *p* < 0.05 vs. OGD group). (**E**) The confocal immunofluorescence staining of Annexin V-mCherry and SYTOX green at 6 h in BV-2 cells of the 4 groups. Scale bar = 20 μm.

At 6 h post-OGD, there was significant increase in the expressions of NLRP3, ASC, and cleaved-caspase-1 when compared to the control group (Figure 5A,B, ** *p* < 0.01, *** *p* < 0.001 vs. CTRL group), while induction of IPC for 10 min could result in marked reduction (^#^ *p* < 0.05, ^##^ *p* < 0.01 vs. OGD group). Moreover, OGD could induce a significant increase in IL-1β (** *p* < 0.01 vs. CTRL group), while IPC treatment reversed its upregulation (Figure 5C, ^#^ *p* < 0.05 vs. OGD group). However, there was not any significant change in the level of IL-18 among different groups (Figure 5D). The results indicate IPC treatment could inhibit NLPR3 inflammasome activation and slightly decrease inflammation at 6 h under OGD state. 

**Figure 6:**
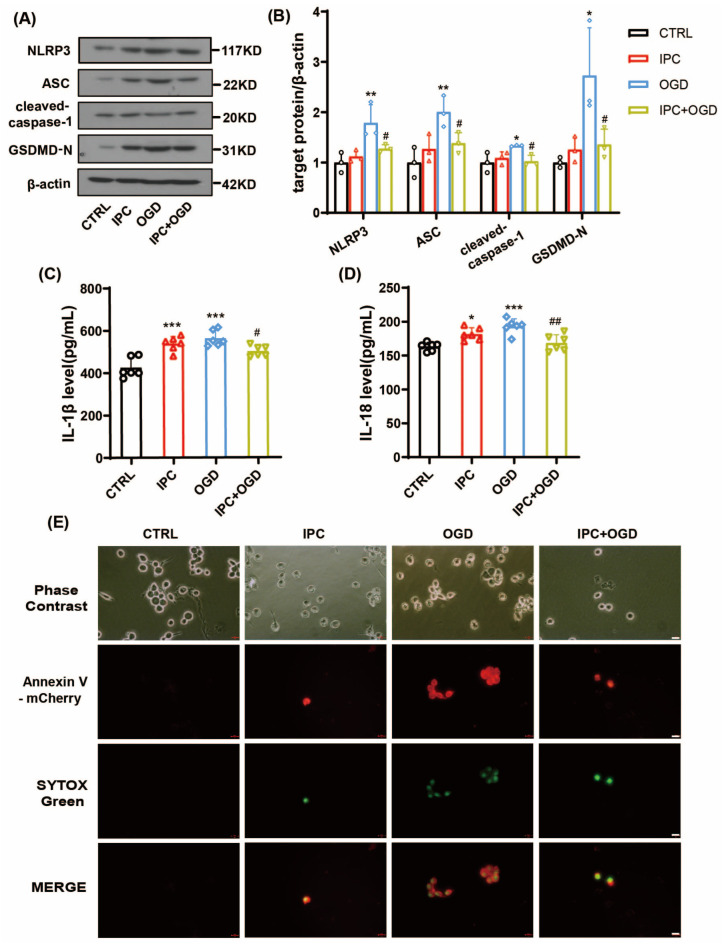
IPC reduced the activation of NLRP3 inflammasome and cell pyroptosis in BV-2 cells at 24 h after OGD. (**A**,**B**) The expressions of NLRP3, ASC, cleaved-caspase-1, and GSDMD-N at 24 h in control, IPC, OGD, and IPC + OGD groups (*n* = 3, * *p* < 0.05, ** *p* < 0.01 vs. CTRL group; ^#^ *p* < 0.05 vs. OGD group). (**C**,**D**) The production of IL-β and IL-18 was assessed by ELISA at 24 h in BV-2 cells of the 4 groups (*n* = 6, * *p* < 0.05, *** *p* < 0.001 vs. CTRL group; ^#^ *p* < 0.05, ^##^ *p* < 0.01 vs. OGD group). (**E**) The confocal immunofluorescence staining of Annexin V-mCherry and SYTOX green at 24 h in BV-2 cells of the 4 groups. Scale bar = 20 μm.

In order to assess the time course of NLRP3 inflammasome activation in vitro, we focused on 24 h recovery from OGD. Accordingly, there was significant upregulation of NLRP3, ASC, and cleaved-caspase-1 at 24 h after OGD (Figure 6A,B, * *p* < 0.05, ** *p* < 0.01 vs. CTRL group), while IPC treatment could obviously decrease the expression of these components (^#^ *p* < 0.05 vs. OGD group). However, unlike 6 h after OGD, the levels of IL-1β (Figure 6C) and IL-18 (Figure 6D) were significantly increased in the IPC-alone group and OGD group (for IL-1β, *** *p* < 0.001 vs. CTRL group; for IL-18, * *p* < 0.05, ** *p* < 0.001 vs. CTRL group), and induction with IPC before OGD significantly inhibited the production of IL-1β and IL-18 when compared with the OGD group (for IL-1β, ^#^ *p* < 0.05 vs. OGD group; for IL-18, ^##^ *p* < 0.01 vs. OGD group).

Taken together, these results suggest that the NLRP3 inflammasome and inflammatory responses are intense at 24 h post-OGD, and that treatment with IPC significantly decreases activation of the NLRP3 inflammasome and inflammatory response, contributing to cell protection after OGD.

### 3.4. IPC Attenuated Cell Pyroptosis after Ischemic Stroke In Vivo and Vitro

To explore whether pyroptosis is involved in the effects of IPC on ischemic stroke, the expression of GSDMD-N was detected under ischemic state in vivo and in vitro. GSDMD-N is the N-domain of GSDMD with membrane pore-forming activity. This can perforate the membrane and induce cell swelling through intracellular contents, releasing proinflammatory mediators to execute pyroptosis and stimulate inflammation [6]. As shown above, the protein level of GSDMD-N was elevated at 6 h (Figure 3A,B, ** *p* < 0.01 vs. Sham group) and 24 h (Figure 4A,B, ** *p* < 0.01 vs. Sham group) after stroke, while IPC reversed its upregulation at both time points (^#^ *p* < 0.05 vs. pMCAO group). Consistent with the findings in vivo, we observed that the expression of GSDMD-N was upregulated at 6 h (Figure 5A,B, *** *p* < 0.001 vs. CTRL group) and 24 h (Figure 6A,B, * *p* < 0.05 vs. CTRL group) under OGD state, but was reversed after IPC treatment at both time points as well (^#^ *p* < 0.05 vs. OGD group). 

To further determine the effects of IPC on cell pyroptosis in vitro, we detected the confocal immunofluorescence staining of Annexin V-mCherry and SYTOX green. Annexin V-mCherry staining was used to assess apoptosis. SYTOX green is a nuclear dye with green fluorescence which can enter cells with a damaged membrane; it was used to assess pyroptosis, as previously reported [18]. The cells undergoing early apoptosis were positive for Annexin V-mCherry staining. In this case, the cells with bright Annexin V-mCherry/SYTOX positive were considered to be dead, as the membranes were permeable to the dye. As shown, we discovered the confocal staining of Annexin V-mCherry/SYTOX-positive cells was increased at 6 h (Figure 5E) and 24 h (Figure 6E) in BV-2 cells subjected to OGD, but IPC could partially attenuate the effects at both time points. Collectively, these data above suggest that IPC may inhibit pyroptosis and attenuate cell death under ischemic conditions. 

## 4. Discussion

In the present study, we conducted an IPC model in vivo and in vitro to explore the relationship between the NLRP3 inflammasome and IPC after ischemic stroke. First, we found that IPC decreased infarct volume and neurological deficits in mice and promoted cell viability under OGD state in BV-2 cells. Then, we discovered that the NLRP3 inflammasome was activated and neuroinflammation was induced under ischemic stress, while IPC could partially inhibit the activation of the NLRP3 inflammasome and suppress the inflammatory response and cell pyroptosis, which provides evidence that IPC may attenuate ischemic injury by inhibiting NLRP3 inflammasome activation and cell pyroptosis. 

To date, the effects of preconditioning have been reported in different organs, such as the brain and kidney, as well as in other tissues. Multiple experimental studies have demonstrated that the brain can be preconditioned to resist prolonged lethal injuries, including ischemia, hypoxia, and trauma. Effective preconditioning stimuli are diverse and comprise brief ischemia, hypoxia, hypothermia, hyperbaric oxygen, and some pharmacological agents. Remote ischemic preconditioning (RIPC), defined as a brief transient ischemia in distant tissues/organs, can protect the remote tissues against subsequent ischemic injuries [3]. Consistent with previous studies, we observed that IPC significantly decreased the infarct volume and attenuated neurological deficits of MCAO mice. Additionally, we found that the time duration affected the effects of IPC under OGD state. Compared to the time duration of 5 min and 15 min, IPC induction with 10 min significantly increased cell viability. Over the past few decades, a wealth of biological mechanisms has been revealed that contribute to the pathogenesis of IPC, involving metabolic dysfunction, a disturbance of the neuronal excitatory/inhibitory balance, activation of extra- and intracellular defense mechanisms, and reduction in inflammatory sequelae [19]. However, the exact mechanisms of IPC remain unknown.

Accumulating evidence has suggested that IPC could modulate immune response and inhibit excessive inflammation at different layers, ranging from molecular, cellular, to systemic mechanisms, to protect against lethal injuries [20]. Some previous studies demonstrated that IPC attenuated ischemic insults via inhibiting nuclear factor kappa B (NF-κB) and cell apoptosis [21,22]. Additionally, IPC was observed to attenuate ischemic injury by suppressing the toll-like receptors (TLRs)/NF-κB signaling pathway [23,24]. Some researches further suggested that RIPC could inhibit neuroinflammation by decreasing the production of IL-1β, IL-6, and interferon-γ under ischemic state [25]. These findings indicate that IPC may reduce inflammatory responses to prevent ischemic injury, while a mechanistic understanding of the inflammatory mediators of IPC may help facilitate transition into the clinical setting.

The NLRP3 inflammasome is a multi-protein complex participating in diverse innate immune processes such as infection, inflammation, and autoimmunity. Once activated, NLRP3 assembles and activates pro-caspase-1, leading to the excretion of inflammatory cytokines and pyroptotic cell death [14]. Microglia are the chief innate immune cells within the brain. In-depth studies have focused on NLRP3 function in microglia. It has been demonstrated that inhibiting NLRP3 activation can mitigate brain damage and disability after ischemic stroke in a wide variety of rodent models and clinical studies [11,12,13,26,27]. Consistently, our findings indicated that the NLRP3 inflammasome was activated in vivo and in vitro under ischemic state as well. However, the role of activated-NLRP3 inflammasome in the process of IPC following ischemic stroke has not been fully understood. Zuurbier et al. observed that the protective effect of IPC was absent in NLRP3-/- hearts in an ex vivo Langendorff-perfused heart model [28]. On the contrast, Pearce et al. demonstrated that RIPC inhibited cytokine release by reducing NF-κB-mediated NLRP3 inflammasome production [29]. Additionally, a recent study reported that hypoxic preconditioning (HPC) suppressed NLRP3 inflammasome activation and prevented cerebral ischemia/reperfusion injury in an ischemic brain [30]. The inconsistent data may be related to the species, models, or paradigms of IPC stimuli used in experimental studies. In the current study, we discovered that IPC could significantly inhibit the expression of NLRP3 inflammasome components and partially reduce the level of IL-1β and IL-18 at 6 h and 24 h following ischemic stroke, especially at the 24 h time point. These findings further provide enhanced evidence to support a key role for innate immune signaling and microglia in IPC-mediated neuroprotection.

Pyroptosis is a proinflammatory form of lytic programmed cell death which can be triggered by extracellular or intracellular stimuli. Morphologically, it is characterized by plasma membrane rupture, followed by release of cellular contents and proinflammatory factors [31]. It has been argued that GSDMD-N, the pore-forming domain of GSDMD, breaks down the permeability of the plasma membrane and initiates pyroptosis [32]. A great deal of evidence indicates that pyroptosis contributes to the pathogenesis of ischemic stroke, while inhibiting pyroptosis may mitigate ischemic brain injury [33,34,35]. Interestingly, Zhou et al. discovered that ischemic-hypoxic preconditioned-olfactory mucosa mesenchymal stem cells may enhance mitochondrial function recovery and suppress neuronal pyroptosis and apoptosis during ischemic stroke [36]. In addition, Pang et al. demonstrated that HPC could decrease the expression of GSDMD and exert neuroprotective effects [30], indicating that cell pyroptosis inhibition contributes to the neuroprotective effects of HPC. In this study, we found that the expression of GSDMD-N was significantly upregulated at 6 h and 24 h after ischemic stroke in vivo and in vitro, together with the increased confocal immunofluorescence staining of Annexin V-mCherry and SYTOX green. However, IPC induction downregulated GSDMD-N expression and reduced the confocal of Annexin V-mCherry and SYTOX green positive cells. These results, together, imply that IPC might inhibit cell pyroptosis and attenuate brain damage during cerebral I/R injury. Nevertheless, these findings must be verified by additional experiments in the future.

This study has some limitations. First, only the dynamic alterations of the NLRP3 inflammasome were investigated after IPC treatment. The relationship between IPC and other inflammasomes, such as NOD-like receptor protein 1 (NLRP1), absent in the melanoma-2 (AIM2) inflammasome, deserves further investigation. Second, we used BV-2 cell lines instead of primary microglia for the in vitro study. BV-2 cell lines could not completely simulate the physiological function of primary microglia. Thus, primary cells should be employed to further explore the underlying mechanisms. Finally, in order to minimize the variables involved, we used only adult male mice for the in vivo experiment. However, stroke clinically occurs primarily in aged individuals. Future experiments should investigate pMCAO in both aged and female mice to increase generalizability of results.

## 5. Conclusions

In summary, we demonstrated that IPC inhibited the activation of the NLRP3 inflammasome and neuroinflammatory responses, suppressed cell pyroptosis, and protected against ischemic injury under ischemic conditions. These findings enhanced the understanding of interplay between IPC and inflammatory signature and cell death, providing novel insights into IPC-mediated neuroprotective effects after ischemic stroke. Exploring the biological mechanisms underlying endogenous neuroprotection as well as validating a novel therapeutic strategy may facilitate direct application of IPC to stroke-related clinical settings. 

## Data Availability

The datasets presented in the study are available from the corresponding authors on reasonable request.
